# Oligo-Metastatic Ductal Breast Carcinoma Presenting as a Subcutaneous Nape Nodule: A Case Report

**DOI:** 10.7759/cureus.71320

**Published:** 2024-10-12

**Authors:** Mitra H Behbehani Pour, Amal Yousif S Abdullah, Mohamed Moneer A Alsayed, Ashraf A Abdeltawab Ibrahim, Aisha N H. S. Y. Alobaidly

**Affiliations:** 1 Department of Surgical Oncology, Kuwait Cancer Control Center (KCCC), Kuwait, KWT

**Keywords:** breast cancer, ductal, kccc, kuwait, nape, oligo-metastatic, oligo-metastatic breast cancer, soft tissue, subcutaneous

## Abstract

Breast cancer (BC) in females is the most diagnosed cancer and the leading cause of cancer death in women worldwide, followed by lung, colorectal, and cervical cancers. For oligo-metastatic (OM) cancers, the best definition is a maximum of five metastatic foci, not necessarily located in the same organ or anatomic region, all potentially treatable by ablative local treatment: either surgical resection or radiation when accessible. Oligo-metastatic breast cancer (OM-BC) is currently arising as an emerging entity with more focused research needed to upgrade the guidelines for best management. Given the heterogeneous nature of BC metastasis, evolving molecular mechanisms have been shown to play a lead way for possible future-guided preventive therapy. Soft tissue metastases are particularly very rare and usually seen arising in subcutaneous fat, fascia, or muscle fibers. They can be confused with a primary soft tissue sarcoma and hence it is important to obtain an accurate differential diagnosis. We hereby present a case of OM-BC presenting as a metastatic soft tissue nodule in the nape in a 73-year-old Kuwaiti lady diagnosed with left breast cancer in 2016 (ductal type) with otherwise free metastatic workup in our practice at Kuwait Cancer Control Center (KCCC).

## Introduction

Since 2021, breast cancer (BC) has become the most common worldwide cancer in women, resembling 12% of all new cancer cases per year, and is considered the leading cause of cancer-associated deaths in women [1]. BC tends to metastasize to multiple distant organs, a phenomenon best known as “metastatic heterogeneity,” with most affected sites usually bones, lungs, liver, brain, and distal lymph nodes [2]. Soft tissue metastasis is extremely rare and can be found in subcutaneous fat or muscle layer; therefore, it should be differentiated from a primary soft tissue sarcoma [3]. Plaza and colleagues published a series of 118 patients with soft tissue metastasis, including only 13 cases from a primary BC. In addition, the authors in the current study did a systematic online literature search and found similar cases with different-site metastasis: soft tissue of the back and left thigh [4,5]. This is probably the first published case of oligo-metastatic breast cancer (OM-BC) (ductal type) to the subcutaneous tissue of the nape of the neck. Although there is still a lack of published data in the field of molecular theories of metastasis, particularly in BC, a deeper understanding is necessary for the potential discovery of promising therapies [6]. In addition, there are not enough solid guidelines about the standard of care for soft tissue metastasis, and the management follows the consensus for oligo-metastatic breast cancer (OM-BC). Therefore, the best management is on a case-by-case basis supported by a multidisciplinary approach. More focused research is required to fulfil the aforementioned areas of interest.

## Case presentation

A 73-year-old lady from Kuwait was diagnosed with left breast cancer, T2 N1 M0, in 2016. Core biopsy revealed invasive ductal carcinoma, grade 2; hormone receptor-positive (estrogen (ER) 95% and progesterone (PR) negative), human epidermal growth factor receptor 2 (HER2) NEU negative and Ki67 was 20%.

In April 2016, she underwent surgery in the form of a left skin-sparing mastectomy (with tissue expander) and axillary clearance. The postoperative histopathological analysis also revealed invasive ductal carcinoma, grade 2, one positive axillary lymph node (1/21), hormone receptor-positive, HER2 NEU negative, and pT2 N1. EndoPredict assay was “high risk 5.3”. She completed adjuvant chemotherapy (12 cycles of weekly paclitaxel and four cycles of dose-dense Adriamycin and Cyclophosphamide) and then kept on endocrinal therapy, letrozole. She underwent further surgery to exchange the expander with a silicone implant, and later, she underwent a total abdominal hysterectomy with bilateral salpingo-oophorectomy due to uterine fibroids.

Recently, she presented with a firm soft tissue nodule with a size of around 2 × 1 cm at the nape of the neck. Fine needle aspiration cytology came positive for carcinoma, mostly metastatic from a primary breast origin. Radiological evaluation of both breasts revealed only benign findings: no evidence of local recurrence or a new primary. 

**Figure 1:**
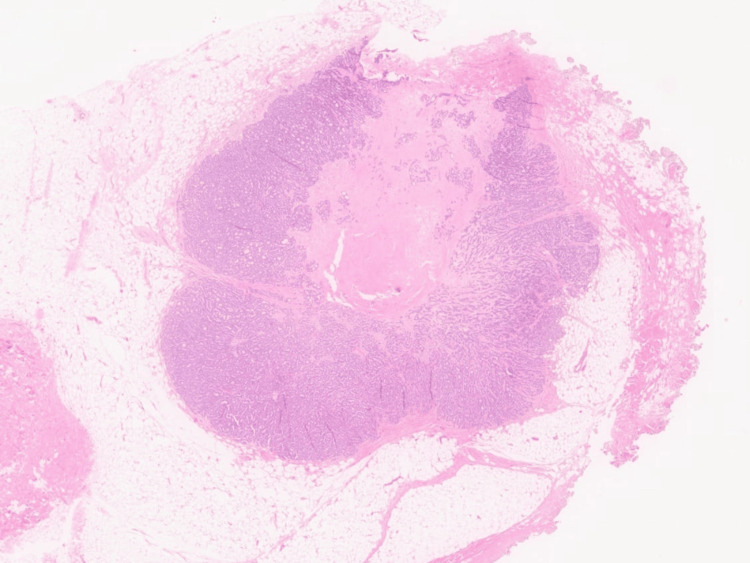
Photomicrograph of tumor within subcutaneous adipose tissue (H&E; 4×). H&E: hematoxylin and eosin.

**Figure 2:**
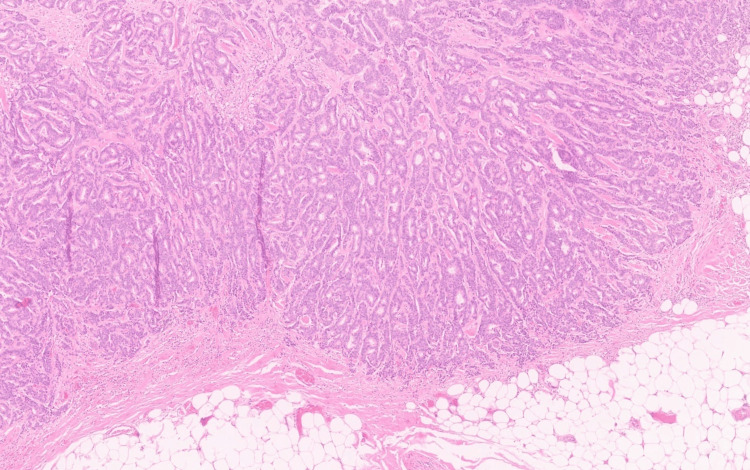
Photomicrograph of the tumor arranged in nests and tubules (H&E; 10×). H&E: hematoxylin and eosin.

**Figure 3:**
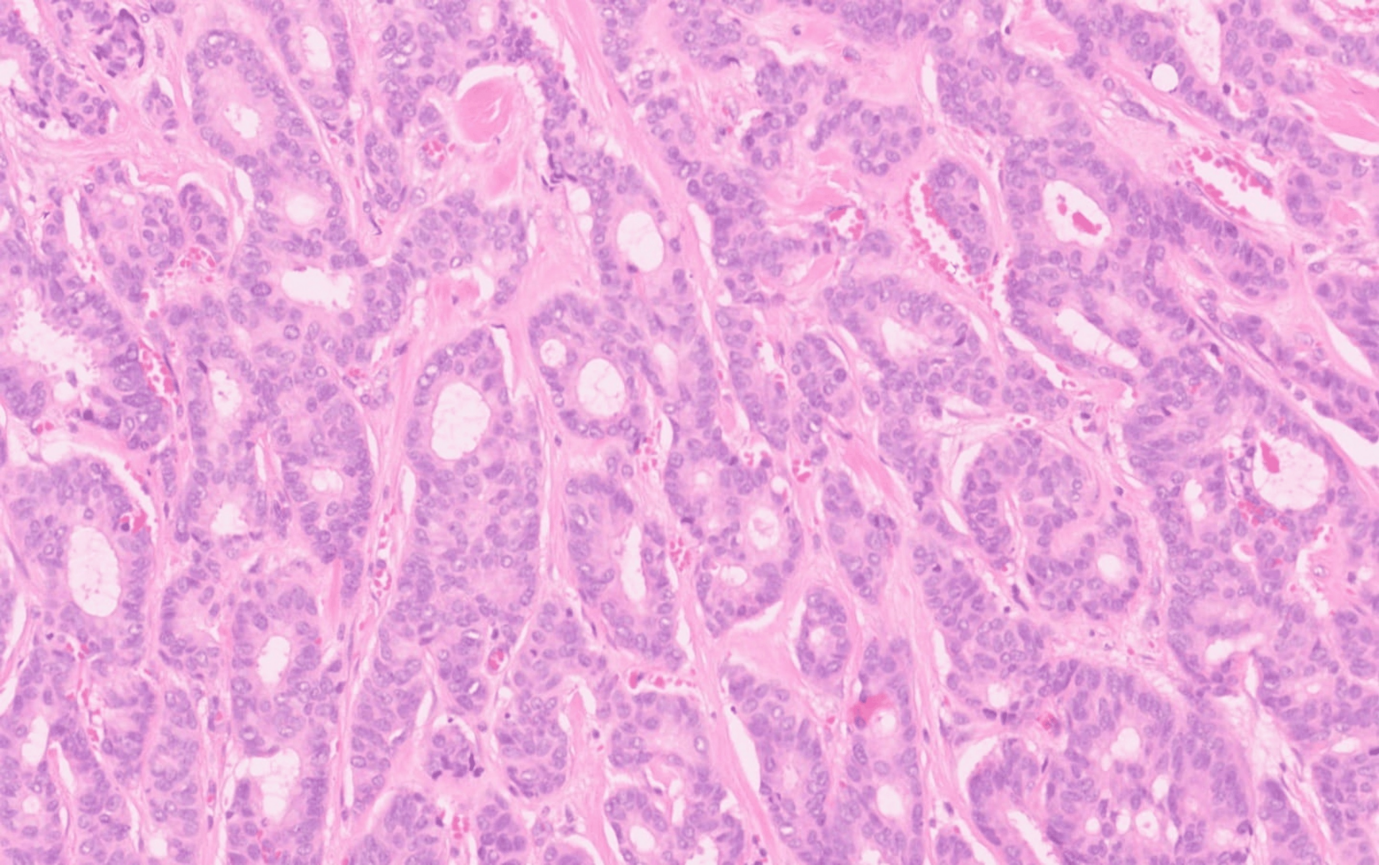
Photomicrograph of the tumor with predominantly tubuloglandular morphology lined by cells with hyper-chromatic nuclei; abundant eosinophilic cytoplasm with brisk mitosis (H&E; 40×). H&E: hematoxylin and eosin.

She underwent further surgery in the form of a wide local excision of the nape lesion in May 2024. Final postoperative histopathology revealed metastatic soft tissue deposits of carcinoma, in keeping with known breast primary. Immunohistochemistry showed hormone receptor positivity (ER 90%, PR 5%) and negative for HER2 NEU. Ki67 was 30%, and tumor cells were positive for pan-cytokeratin (CK), CK7, and E-cadherin. Margins were free of tumors, and the overlying skin was unremarkable. Further metastatic workup was unremarkable; the case was confirmed to have an oligo-metastatic nape nodule. She discussed afterward in a multidisciplinary team (MDT) meeting, and the consensus was no need for postoperative radiotherapy as the lesion was entirely excised, the patient was to be kept on endocrine therapy (letrozole) and regular follow-up. Figures 1-3 show the tumor cells appearing in the resected specimen.

## Discussion

Metastatic soft tissue masses secondary to a primary carcinoma are uncommon and can be confused clinically, and even more histologically, with primary soft tissue sarcomas. Therefore, it is of paramount importance to differentiate between both entities because treatment and prognosis are not similar [7,8]. For BC, up to 30% of cases can develop metastases even years after definitive management of the primary tumor. In addition, up to 90% of deaths are metastasis related. To date, the reported incidence of soft tissue metastasis from a primary BC is 0.2-0.8% [1,7]. The theory behind this rarity assumes that soft tissues tend to deter metastatic invasion by producing anticancer factors like protease inhibitors, lactic acid, and possibly the presence of beta-adrenergic receptors. According to the authors' literature search, similar cases were recorded with soft tissue metastasis to the chest wall, shoulder; scapula, back, lower back (right lumbar region), and left thigh [3-5]. BC is a heterogeneous disease with a complex molecular mechanism of metastatic tendency. Some key proteins were identified to play a major role in the process of metastasis, including integrins, which are involved in tumor cellular survival and motility; E-cadherin, responsible for cell-to-cell adhesion; transforming growth factor-β (TGFβ) linked to epithelial-to-mesenchymal transformation (EMT), leading to increased metastatic invasion potential; and vascular endothelial growth factor (VEGF), responsible for tumor angiogenesis, and this is particularly important because it can give rise to a molecular-guided therapy to help reduce metastatic potential in patients with BC [6]. This particular area is evolving, and further research is recommended.

Around 95% of BC are adenocarcinomas arising at the terminal duct lobular unit, with invasive ductal carcinoma being the most common subtype (50-80%), followed by invasive lobular carcinoma (5-15%), then medullary (5-7%) and mucinous (2%) carcinomas [1,7].

Around 60-80% of BC cases are positive for ER and PR expression. ER-positive patients tend to have a better response to endocrinal treatment, have longer survival, and have a lower rate of recurrence in contrast to ER-negative or ER- and PR-negative ones. Therefore, patients with stage IV or recurrent disease who are ER/PR positive, and HER-2 negative without visceral metastasis are usually treated with endocrinal therapy alone or in combination with targeted agents. Aromatase enzyme inhibitors such as anastrozole are proven to have equivalent efficacy and lower rates of side effects compared to tamoxifen for postmenopausal patients [1,8-10]. Recent studies are suggesting a promising role of cyclin-dependent kinase (CDK) 4/6 inhibitors in the treatment of OM-BC, even with possible curative intent [11], but this has to be supported by further research to obtain a solid consensus.

The five-year overall survival rate in BC patients without metastasis can reach higher than 80%. However, metastatic breast cancer (MBC) decreases this rate significantly to around 25% [12,13]. The time interval between the primary diagnosis and/or treatment of BC and the occurrence of distant metastases can reach up to 15 years, according to a recently published case report [14]. MBC was traditionally considered incurable since treatment options, despite aiming to prolong survival, are based on a palliative concept. This has remained unchallenged for a long time; however, the uprising attention to the pattern of OM-BC is now changing this concept. Indeed, around 20% of patients with MBC present with OM-BC, meaning MBC at presentation (de novo stage IV) or recurrence with low metastatic burden. Advances in radiological modalities, having increased sensitivity, together with the availability of least invasive loco-regional treatments, added to a crescendo-pattern of patients’ expectations, are all contributing to paying more attention to the definition of OM-BC, and more research is needed to refine the standard of care for this emerging category [2,11,15].

## Conclusions

In all patients with a history of breast cancer, distant metastasis should be ruled out even years after diagnosis and treatment. Soft tissue metastases are very rare, and this is probably the first case in literature to be recorded to have isolated oligo-metastasis in the subcutaneous tissue of the nape of the neck from a primary breast origin, of ductal subtype, after an interval of eight years. More focused research is needed to refine the standard of care for this particular entity of OM-BC, especially soft tissue metastases. In addition, more understanding of the role of molecular mechanisms in MBC is needed, as the rarity of recorded cases is not enough to establish solid management guidelines. In addition, a multidisciplinary approach is of paramount importance on a case-by-case basis.

## References

[1] (2024). Breastcancer.org. Breast cancer facts and statistics. http://www.breastcancer.org/facts-statistics.

[2] Liang Y, Zhang H, Song X, Yang Q (2020). Metastatic heterogeneity of breast cancer: molecular mechanism and potential therapeutic targets. Semin Cancer Biol.

[3] Plaza JA, Perez-Montiel D, Mayerson J, Morrison C, Suster S (2008). Metastases to soft tissue: a review of 118 cases over a 30-year period. Cancer.

[4] Salwa F, Maha A (2017). Soft tissue metastasis of breast cancer. Cancer Ther Oncol.

[5] Melissa R, Alexieva J, Coogan A, Hussein L, Marcus E, Wecsler J (2024). Abstract PO5-20-07: a case of metastatic breast cancer to soft tissue of the thigh. Cancer Res.

[6] Tungsukruthai S, Petpiroon N, Chanvorachote P (2018). Molecular mechanisms of breast cancer metastasis and potential anti-metastatic compounds. Anticancer Res.

[7] Leinung S, Möbius C, Udelnow A, Hauss J, Würl P (2007). Histopathological outcome of 597 isolated soft tissue tumors suspected of soft tissue sarcoma: a single-center 12-year experience. Eur J Surg Oncol.

[8] Gradishar WJ, Anderson BO, Balassanian R (2018). Breast cancer, version 4.2017, NCCN clinical practice guidelines in oncology. J Natl Compr Cancer Netw.

[9] Yang G, Lu T, Weisenberger DJ, Liang G (2022). The multi-omic landscape of primary breast tumors and their metastases: expanding the efficacy of actionable therapeutic targets. Genes (Basel).

[10] Buzdar AU (2001). Endocrine therapy in the treatment of metastatic breast cancer. Semin Oncol.

[11] Valente A, Teixeira Tavares N, Caeiro C, Barbosa M, Augusto I (2023). The role of cyclin-dependent kinase 4/6 inhibitors treatment in oligometastatic breast cancer: a case report on a possible curative intent strategy. Cureus.

[12] Gibson L, Lawrence D, Dawson C, Bliss J (2009). Aromatase inhibitors for treatment of advanced breast cancer in postmenopausal women. Cochrane Database Syst Rev.

[13] (2024). International Agency for Research on Cancer. Cancer today. https://gco.iarc.fr/today/en/dataviz/pie.

[14] Palacios Huatuco RM, Ramírez MF, Stoppani I, Mendoza Santos H, Mayer HF (2023). Metastasis of invasive ductal breast cancer in the subcutaneous tissue of the back: report of a relapse at 15 years. Int J Surg Case Rep.

[15] Miglietta F, Bottosso M, Griguolo G, Dieci MV, Guarneri V (2022). Major advancements in metastatic breast cancer treatment: when expanding options means prolonging survival. ESMO Open.

